# 总补体活性对阵发性睡眠性血红蛋白尿症患者溶血及依库珠单抗疗效的意义

**DOI:** 10.3760/cma.j.cn121090-20250118-00034

**Published:** 2025-09

**Authors:** 玲 李, 潇弋 黄, 晓庆 丁, 紫薇 刘, 辰 杨, 苗 陈, 剑 殷, 冰 韩

**Affiliations:** 1 北京中医药大学东方医院，北京 100078 Dongfang Hospital of Beijing University of Chinese Medicine, Beijing 100078, China; 2 中国医学科学院北京协和医学院北京协和医院，北京 100730 Peking Union Medical College Hospital, Peking Union Medical College, Chinese Academy of Medical Sciences, Beijing 100730, China; 3 北京医院，北京 100730 Beijing Hospital, Beijing 100730, China

## Abstract

回顾性收集2023年1月至2024年6月北京协和医院、北京中医药大学东方医院收治的阵发性睡眠性血红蛋白尿症（PNH）患者数据，选择经过足量依库珠单抗治疗至少3个月，并完成了依库珠单抗治疗前、治疗后3个月的总补体活性（CH50）水平检测的25例患者，其中24例患者完成了6个月治疗及CH50检测。依库珠单抗治疗3及6个月后，所有PNH患者症状显著改善，乳酸脱氢酶（LDH）从基线（1 814.4±924.8）U/L下降至（248.5±61.0）U/L和（239.3±44.8）U/L。HGB水平从基线（73.9±14.4）g/L上升至（99.9±21.3）g/L和（99.6±19.8）g/L。CH50基线水平为（32.4±14.7）％，治疗后3和6个月分别下降至2.0％（1.0％～8.0％）和1.0％（1.0％～4.0％）。基线时CH50水平与LDH呈线性相关（*P*<0.001，*r*＝0.789），且CH50水平在依库珠治疗后3和6个月与LDH均明显下降，变化趋势相似，但在用药3及6个月的CH50水平与LDH及其他参数不存在线性相关。提示血清CH50水平可能是依库珠单抗诱导的补体阻断的标志物，可一定程度上反映PNH血管内溶血及依库珠单抗疗效。

## Abstract (en)

This study retrospectively analyzed data from 25 patients with paroxysmal nocturnal hemoglobinuria（PNH）admitted to Peking Union Medical College Hospital and Dongfang Hospital of Beijing University of Chinese Medicine from January 2023 to June 2024. Patients receiving sufficient eculizumab treatment for at least 3 months and who completed hemolytic complex（CH50）level testing pre- and post-treatment for 3 and 6 months were selected. Blood routine, biochemistry, and the 50％ CH50-related indicators were monitored pre- and post-treatment. Among these patients, 24 completed 6 months of treatment and CH50 testing. After 3 and 6 months of eculizumab treatment, all patients with PNH showed significant improvement in symptoms, with lactate dehydrogenase（LDH）levels decreasing from a baseline of（1 814.4±924.8）U/L to（248.5±61.0）U/L and（239.3±44.8）U/L. Hemoglobin levels increased from a baseline of（73.9±14.4）g/L to（99.9±21.3）g/L and（99.6±19.8）g/L. The baseline CH50 level was（32.4±14.7）％, which decreased to 2.0％（1.0％–8.0％）and 1.0％（1.0％–4.0％）at 3 and 6 months posttreatment, respectively. At baseline, a linear correlation was found between CH50 and LDH levels（*P*<0.001）, and the trend of CH50 changes was significantly lower than LDH at 3 and 6 months post-treatment with eculizumab, with similar trends. However, no linear correlation was observed between CH50 and LDH levels or other parameters at 3 and 6 months of medication. Our case demonstrates that eculizumab is effective for PNH hemolysis treatment. The serum CH50 level may be a biomarker for complement blockade induced by eculizumab, which can, to some extent, reflect the intravascular hemolysis of PNH and the efficacy of eculizumab.

阵发性睡眠性血红蛋白尿症（Paroxysmal nocturnal hemoglobinuria, PNH）是一种造血干细胞基因突变导致克隆缺陷而发生的溶血性疾病[1]。补体调节蛋白在红细胞表面丢失，导致红细胞对补体敏感性增加，发生血管内溶血[1]–[2]，其溶血发生过程与补体作用密切相关。依库珠单抗通过阻断末端补体C5活化，抑制血管内溶血，极大改善了PNH患者生活质量、提升了PNH患者存活率，被各大指南推荐作为治疗溶血性PNH的首选药物[3]–[5]。

近年来国外研究发现，血清总补体活性（The 50％ haemolytic complement, CH50）可通过测量诱导抗体致敏绵羊红细胞50％裂解所需血浆量，来反映终末及经典补体活性[6]–[7]，评估补体的整体功能，可作为监测依库珠单抗疗效的标志物[8]–[9]，但国内尚缺乏该方面的研究。

为阐明CH50在反映PNH溶血活性、依库珠单抗治疗中溶血活性监测的临床意义，我们回顾性收集了部分接受足量依库珠单抗治疗至少3个月，且进行了治疗前及治疗后3个月CH50检测的病例资料，分析CH50与疾病治疗前后溶血情况及依库珠单抗疗效的关系。

## 病例与方法

一、研究对象

本研究为回顾性队列研究，收集自2023年1月至2024年6月在北京协和医院、北京中医药大学东方医院规律使用依库珠单抗至少3个月，且至少进行了治疗前、治疗后3个月CH50检测的PNH患者资料。PNH的诊断和分类参照国际PNH兴趣小组（International PNH interest group, IPIG）标准[10]。纳入分析的患者需同时满足以下条件：①符合《阵发性睡眠性血红蛋白尿症诊断与治疗中国指南（2024年版）》诊断标准[5]；②年龄≥12岁；③使用依库珠单抗前存在血管内溶血的临床表现或并发症，且患者乳酸脱氢酶（Lactate dehydrogenase, LDH）≥1.5倍正常上限（Upper limit of normal, ULN）；④足量使用依库珠单抗周期治疗至少3个月；⑤至少进行了依库珠单抗治疗前及治疗后3个月CH50监测；⑥患者临床病历材料齐全；⑦本人或监护人签署知情同意书，愿意提供相关的临床数据。

二、研究方法

1. 临床资料收集：统计纳入患者人口学信息、病史（包括血栓形成史、输血史）、体格检查、基线实验室检查指标［包括血常规、网织红细胞计数（Reticulocyte, Ret）、LDH、总胆红素（Total bilirubin, TBIL）、血清铁蛋白（Serum ferritin, SF）、肌酐（Creatinine, Cr）等］、除依库珠单抗外的其他相关治疗情况、CH50水平、骨髓穿刺/活检结果等。依库珠单抗治疗期间，除骨髓穿刺/活检结果外，前3个月每月、随后每3个月收集上述数据。

2. 依库珠单抗用药情况：所有患者在接受依库珠单抗治疗前至少两周接种脑膜炎双球菌疫苗，依库珠单抗前4周每7 d输注600 mg，第5周起每14 d输注900 mg。

3. CH50水平检测：静脉穿刺采集外周血4～5 ml，置于EDTA抗凝管中，将其中一份静脉血标本置于常温下，使用低速冷冻离心机（型号008AS，日本日立）进行离心（离心半径4 cm，转速3 000 r/min，时间20 min），−20 °C保存待检。应用酶联免疫试剂盒（型号AF651，日本和光）检测血清CH50水平。既往研究表明，使用一种存储于−80 °C下的具有100％ CH50活性的标准血浆池作为内部参考标准，CH50活性<10％意味着测试血浆样本相对于标准血浆池（即CH50＝100％）具有<10％的补体活性，即测试者CH50水平<10％时可认为该测试者补体处于阻断状态[7],[11]。

4. 疗效判定标准及安全性分析：记录治疗前及治疗后不同时间点的临床表现；记录用药后3个月和6个月HGB、LDH及其余各项实验室检查指标变化；记录用药后3个月和6个月CH50水平变化；采用FACIT-Fatigue评分评估患者用药前后疲劳程度[12]；记录突破性溶血（Breakthrough hemolysis, BTH）[13]、血管外溶血（Extra vascular hemolysis, EVH）[14]的比例，输血依赖患者脱离输血依赖的比例；记录不良反应，不良反应参考国际通用不良反应分级（Common terminology criteria for adverse events, CTCAE）5.0版。

5. 统计学处理：采用SPSS 26.0和R 4.5.0软件进行数据处理。计数资料采用例数（构成比）表示，组间比较采用卡方检验或Fisher确切概率法。定量资料先进行Shapiro-Wilk正态性检验，对于符合正态分布的定量资料以*x*±*s*描述，对于不符合正态分布的定量资料用中位数（范围）描述；不同指标用药后与基线比较根据Shapiro-Wilk正态性检验结果，采用配对样本*t*检验或配对Wilcoxon秩和检验进行差异性比较，采用Spearman法进行相关性分析并用R 4.5.0绘制相关分析矩阵图。双侧*P*<0.05为差异有统计学意义。

## 结果

一、患者临床特征

本研究共纳入25例患者，其中男11例（44.0％），女14例（56.0％）。中位年龄41（25～79）岁。经典型PNH患者10例（40.0％），PNH合并再生障碍性贫血（AA）患者15例（60.0％）。所有患者在使用依库珠单抗前，均存在血红蛋白尿发作，每月发作次数为3（1～10）次，用药前的FACIT-Fatigue评分（25.3±12.2）分。输血依赖10例（40.0％）；合并血栓4例（16.0％），分别为肠系膜血栓（2例）、肝静脉血栓（1例）和下肢深静脉血栓（1例）；肾功能损害2例（8.0％）。25例患者基线CH50为（32.4±12.7）％，LDH为（1 814.4±924.8）U/L，其余血常规、生化等实验室指标见表1。

**表1:** 阵发性睡眠性血红蛋白尿症（PNH）患者基线及依库珠单抗治疗不同时间各指标比较

临床特征	基线（25例）	3个月（25例）	*P*_1_值	6个月（24例）	*P*_2_值
LDH（U/L, *x*±*s*）	1 814.4±924.8	248.5±61.0	<0.001	239.3±44.8	<0.001
HGB（g/L, *x*±*s*）	73.9±14.4	99.9±21.3	<0.001	99.6±19.8	<0.001
Ret#［×10^9^/L, *M*（范围）］	189.8（66.5～531.4）	233.3（94.0～379.4）	0.690	216.1（118.7～397.7）	0.541
Ret％［％, *M*（范围）］	8.9（1.7～27.1）	7.2（2.5～19.3）	0.230	8.8（2.9～19.4）	0.362
WBC［×10^9^/L, *M*（范围）］	3.2（1.4～8.2）	2.9（1.1～8.9）	0.108	3.2（1.6～10.9）	0.191
PLT［×10^9^/L, *M*（范围）］	123.5（51～459）	124（27～375）	0.424	117（68～280）	0.152
SGPT［U/L, *M*（范围）］	15.6（4.3～44.3）	14.1（5.3～61.8）	0.424	18.1（7.4～52.1）	0.307
TBIL［µmol/L, *M*（范围）］	29.0（9.6～75.9）	31.6（12.9～71.7）	0.798	31.7（15.2～67.6）	0.839
DBIL［µmol/L, *M*（范围）］	12.9（4.8～30.8）	12.0（5.5～38.5）	0.989	14.4（6.7～32.1）	0.152
Cr［µmol/L, *M*（范围）］	74（36～371）	74（42～230）	0.152	81.5（64～128）	0.241
SF［mg/L, *M*（范围）］	20.2（6.0～678.0）	27.1（8.3～484.3）	0.815	158.1（11.4～467.0）	0.146

**注** LDH：乳酸脱氢酶；Ret#：网织红细胞绝对值；Ret％：网织红细胞百分比；SGPT：血清丙氨酸转氨酶；TBIL：总胆红素；DBIL：间接胆红素；Cr：血肌酐；SF：血清铁蛋白。*P*_1_：依库珠单抗治疗3个月与基线比较；*P*_2_：依库珠单抗治疗6个月与基线比较

依库珠单抗治疗前用药包括：14例（56.0％）长期使用糖皮质激素，8例（32.0％）使用雄激素，8例（32.0％）使用环孢素A，2例（8.0％）使用海曲泊帕，2例（8.0％）使用利伐沙班，4例（16.0％）未治疗。开始依库珠单抗治疗后，使用糖皮质激素者全部逐渐停用糖皮质激素，5例停用雄激素，3例停用环孢素A，1例停用海曲泊帕，其余患者均未改变原有治疗方案。

所有患者都完成了基线CH50及血常规、生化等实验室指标检测。所有患者完成了至少3个月的依库珠单抗治疗及3个月的CH50检测，其中24例患者完成了6个月依库珠单抗治疗及6个月的CH50检测。

二、疗效

依库珠单抗治疗的中位时间为6（3～12）个月，随访时间为治疗后5（3～12）个月。至随访期末，25例患者血红蛋白尿每月发作次数为0（0～2）次（*P*<0.001）；随访期间所有患者未见新发血栓形成（*P*＝0.043）。使用依库珠单抗前10例存在输血需求的患者，在用药2周后均摆脱输血依赖（*P*＝0.001）。2例伴肾功能损害患者在用药期间肌酐水平均明显下降。用药前的FACIT-Fatigue评分为（25.3±12.2）分。用药3个月及随访末FACIT-Fatigue评分分别为（40.4±7.4）分和（42.3±7.4）分，疲劳程度较基线水平显著改善（*P*<0.001）。

依库珠单抗治疗3、6个月后，25例患者LDH水平从基线（1 814.4±924.8）U/L下降至（248.5±61.0）U/L和（239.3±44.8）U/L，HGB水平从基线（73.9±14.4）g/L上升至（99.9±21.3）g/L和（99.6±19.8）g/L。其他血液学参数较基线治疗前后差异均无统计学意义（均*P*>0.05），具体见表1。

三、依库珠单抗治疗前后CH50的变化及与溶血和疗效的关系

依库珠单抗用药前，25例患者的CH50为（32.4±14.7）％，治疗后3个月和6个月的CH50分别为2.0％（1.0％～8.0％）和1.0％（1.0％～4.0％），与基线相比，CH50水平显著降低（均*P*<0.001）；依库珠单抗治疗前、后CH50的变化趋势与LDH变化趋势相似。分别将基线（25例）、3个月（25例）和6个月（24例）的CH50与该时点下对应的血常规、生化等指标进行相关性分析，并绘制相关分析矩阵。结果显示，基线时CH50与LDH呈线性正相关（*P*<0.001，*r*＝0.789），但与血常规、Ret及其他生化指标无统计学相关（均*P*>0.05）（图1A）。用药3、6个月后，尽管CH50和LDH均较基线显著下降，但CH50与LDH不存在线性相关性（*P*值分别为0.338、0.577）。同时。用药后3个月及6个月的CH50与血常规、其他生化指标也无统计学相关（均*P*>0.05）（图1B、C）。

**图1:**
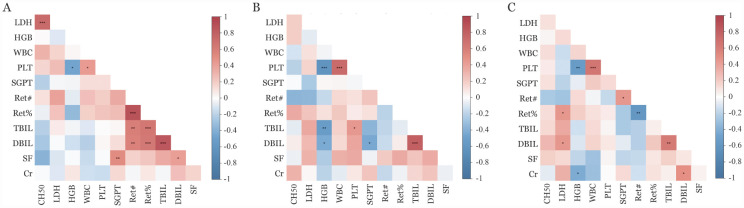
PNH患者CH50与血常规、生化等指标Spearman相关性矩阵图（*P<0.05、**P<0.01、***P<0.001） **A** 基线；**B** 依库珠单抗用药3个月后；**C** 依库珠单抗用药6个月后 **注** PNH：阵发性睡眠性血红蛋白尿症；CH50：总补体活性；LDH：乳酸脱氢酶；SGPT：血清丙氨酸转氨酶；Ret#：网织红细胞绝对值；Ret％：网织红细胞百分比；TBIL：总胆红素；DBIL：直接胆红素；SF：血清铁蛋白；Cr：肌酐

四、不良反应、BTH及EVH

6例（24.0％）患者用药后出现头痛，对症处理后3 d内缓解；6例（24.0％）患者出现上呼吸道感染，经对症治疗后于1周内痊愈；1例（4.0％）患者发生肺部感染，经对症治疗后好转；无其他不良事件，无不良反应导致停药的情况，无死亡事件。

BTH：1例（4.0％）患者在用药前CH50 12％、LDH 693.4 U/L，用药3个月后CH50 1％、LDH 144.3 U/L，用药6个月末因肺部感染发生BTH，出现明显血红蛋白尿，急查CH50及血常规、生化，HGB水平从感染前>90 g/L下降至63 g/L，LDH水平由167.3 U/L升高至829.7 U/L，CH50由3％升高至7％，予抗感染治疗患者感染好转后，血红蛋白尿停止，继续行依库珠单抗规律治疗，LDH降至247.3 U/L，CH50降至2％。

EVH：2例（8.0％）患者经依库珠治疗3个月后，LDH水平降至<1.5 ULN，CH50由18％、9％均降至1％。但Ret#、Ret％较基线均上升，且HGB均波动在60.0～80.0 g/L，较基线未见明显上升，疲劳症状改善不明显，Coombs试验（++），诊断为EVH。在进行EVH诊断后，再次对该2例患者各项指标及CH50测定，CH50仍均为1％，LDH分别为282.3 U/L和265.6 U/L。

## 讨论

PNH的血管内溶血活跃程度可通过LDH进行判断[15]。然而，LDH升高受到肿瘤、手术、用药等因素的影响[16]–[17]。由于未经治疗的PNH本质上是一种补体激活引起的疾病，因此CH50在监测PNH溶血机制上特异性会优于LDH，可从总补体激活程度方面判断患者血管内溶血情况。在上述通过LDH评估血管内溶血受限的情况下，同时检测LDH、CH50水平，可以更好提示补体系统活性变化。

既往研究表明，CH50能够反映补体C1～C9等9种成分的综合补体活性水平，对经典途径的血清补体成分减少、缺失和（或）不活跃都很敏感[18]–[20]。依库珠单抗能阻断补体终末途径，抑制血管内溶血发生，但也存在不能完全改善贫血，或者面临BTH等问题[21]。国外的文献报道，对于使用C5补体抑制剂的患者来说，CH50测定可能可以反映因补体活性控制不佳或异常激活[7]，而中国尚未开展相关的检测。

本研究纳入的25例PNH患者，在依库珠单抗用药前，LDH均值约为7.3 ULN，相对应的CH50为（32.4±14.7）％。依库珠单抗治疗后的3和6个月，除1例患者在出现BTH后，LDH再次短暂升高外，其余患者LDH均<1.5 ULN，疲乏量表FACIT-Fatigue评分、HGB较基线均明显提升，相对应的，CH50水平较基线明显下降（均*P*<0.001）。Lundberg等[22]的样本基线CH50水平>45.0％（*n*＝202），而Peffault de Latour一项研究显示，存在溶血事件的PNH患者基线CH50为（19.7±27.6）％（*n*＝19）[23]，我们的患者基线CH50数值介于不同研究之间，但均高于正常值，这可能与不同实验室的实验条件、正常值标准有关。

我们随后分析了CH50与血常规、部分生化指标的关系。基线CH50仅与LDH呈线性相关（*P*<0.001，*r*＝0.789），而与其他参数无关。Peffault de Latour等[7]在分析184例接受依库珠单抗治疗的PNH患者血清样本后，也观察到LDH与CH50存在统计学关联（平均LDH每增加100 U/L，CH50增加1.4％），他们同样没有发现CH50与HGB、网织红细胞存在统计学关系，与我们的结果一致。

但我们在用药3、6个月后，并未发现CH50与血常规、生化指标存在线性相关（均*P*>0.05），然而，CH50的下降，对应了LDH的下降，在后续BTH的患者中，我们也看到CH50与LDH变化的相似趋势，这一定程度上反映了血管内溶血中CH50与LDH变化的可能联系。目前国内外尚未有此方面的研究。

与血常规及LDH等常规检测指标相比，CH50水平在监测PNH患者用药过程中具有以下优势：①由于补体通路形成的C5b-9膜攻击复合物破坏红细胞是PNH最重要的溶血机制，CH50能够针对补体相关溶血机制直观反应溶血程度从而评估PNH病情严重情况；②对使用终末补体抑制剂治疗的PNH患者疗效评估指标如HGB、LDH的影响因素非常多，而CH50对于补体途径介导的血管内溶血监测特异性较强；③CH50水平检测灵敏度高，可直观反映补体活性的变化，明确溶血发生是否由补体介导[24]。

虽然例数较少，我们认为CH50与BTH可能存在一定相关性。本研究1例BTH病例，基线LDH、CH50均在较高水平，依库珠单抗治疗后，LDH<1.5 ULN、CH50<10％。而出现BTH时，在LDH再次>2 ULN、HGB下降的同时，CH50再次上升，当BTH得到控制后，CH50也回落至用药期间水平。本例BTH患者提示，监测CH50的变化，可能也可以为BTH提供佐证，但还需要更多数据支持。

而CH50与EVH则关系不明确。Subías Hidalgo等[25]发现经依库珠单抗用药的PNH患者CH50<10％时仍存在残余持续溶血，且这种溶血可能与EVH有关。在我们研究中，2例出现EVH的患者CH50、LDH水平与诊断EVH之前均无明显变化，我们认为CH50、LDH在反映EVH程度时并不灵敏。McKinley等[26]在寻找EVH的敏感参数时也发现LDH并不能很好地反映EVH程度，这可能与EVH红细胞破坏后被肝脾中巨噬细胞吞噬相关。国外研究认为，监测PNH生物标志物，应考虑末端和近端补体抑制剂之间作用模式的差异，CH50水平测定不能检测到补体系统的细微变化[27]–[28]。

本研究是回顾性研究，可能存在选择偏倚、部分临床数据不完整、样本量小等问题，后续应扩大样本量、延长随访时间以验证初步发现。但我们在国内首先建立了CH50监测PNH溶血及依库珠单抗治疗后溶血控制的情况，并阐明了其与LDH的相关性，为后续进一步完善相关检查，提供一定的参考依据。
